# Relationship between lower-limb muscle strength and functional independence among elderly people according to frailty criteria: a cross-sectional study

**DOI:** 10.1590/1516-3180.2014.1325669

**Published:** 2014-07-22

**Authors:** Fernanda Sotello Batista, Grace Angélica de Oliveira Gomes, Maria José D'Elboux, Fernanda Aparecida Cintra, Anita Liberalesso Neri, Maria Elena Guariento, Maria da Luz Rosário de Souza

**Affiliations:** I MSc. Physiotherapist, Municipal Health Department, Santos, São Paulo, Brazil; II PhD. Professor, Department of Gerontology, Universidade Federal de São Carlos (UFSCar), São Carlos, São Paulo, Brazil; III PhD. Professor, School of Nursing, Universidade Estadual de Campinas (Unicamp), Campinas, São Paulo, Brazil; IV PhD. Full Professor, Department of Education, Universidade Estadual de Campinas (Unicamp), Campinas, São Paulo, Brazil; V PhD. Professor, Department of Internal Medicine, Faculdade de Ciências Médicas, Universidade Estadual de Campinas (Unicamp), Campinas, São Paulo, Brazil; VI PhD. Professor, Department of Dentistry, Faculdade de Odontologia de Piracicaba (FOP), Universidade Estadual de Campinas (Unicamp), Piracicaba, São Paulo, Brazil

**Keywords:** Elderly, Muscle strength, Disability evaluation, Activities of daily living, Frail elderly, Idoso, Força muscular, Avaliação da deficiência, Atividades cotidianas, Idoso fragilizado

## Abstract

**CONTEXT AND OBJECTIVE:**

Muscle strength and functional independence are considered to be determinants of frailty levels among elderly people. The aim here was to compare lower-limb muscle strength (LLMS) with functional independence in relation to sex, age and number of frailty criteria, and to ascertain the influence of these variables on elderly outpatients' independence.

**DESIGN AND SETTING:**

Quantitative cross-sectional study, in a tertiary hospital.

**METHODS:**

The study was conducted on 150 elderly outpatients of both sexes who were in a cognitive condition allowing oral communication, between October 2005 and October 2007. The following instruments were used: five-times sit-to-stand test (FTSST), Functional Independence Measurement (FIM) and Lawton's Instrumental Activities of Daily Living Scale (IADL). Descriptive, comparative, multivariate, univariate and Cronbach alpha analyses were performed.

**RESULTS:**

The mean time taken in the FTSST was 21.7 seconds; the mean score for FIM was 82.2 and for IADL was 21.2; 44.7% of the subjects presented 1-2 frailty criteria and 55.3% > 3 criteria. There was a significant association between LLMS and functional independence in relation to the number of frailty criteria, without homogeneity regarding sex and age. Functional independence showed significant influence from sex and LLMS.

**CONCLUSION:**

Elderly individuals with 1 or 2 frailty criteria presented greater independence in all FTSST scores. The subjects with higher LLMS presented better functional independence.

## Abstract (pt)

**CONTEXTO E OBJETIVO:**

A força muscular e a independência funcional são consideradas indicadores de níveis de fragilidade em idosos. O objetivo foi comparar a força muscular de membros inferiores (FM de MMII) com a independência funcional em função de sexo, idade e número de critérios de fragilidade e verificar a influência dessas variáveis na independência em idosos ambulatoriais.

**TIPO DE ESTUDO E LOCAL:**

Estudo transversal quantitativo, em hospital terciário.

**MÉTODOS:**

Estudo realizado entre outubro de 2005 e outubro de 2007 com 150 idosos ambulatoriais de ambos os sexos, com condições cognitivas suficientes para comunicação oral, sendo utilizados os seguintes instrumentos: teste de levantar e sentar da cadeira cinco vezes consecutivas, Medida de Independência Funcional (MIFm) e Escala de Atividades Instrumentais (AIVDt) de Lawton. Foram realizadas análises descritivas, de comparação, multivariadas, univariadas e de alfa de Cronbach.

**RESULTADOS:**

O tempo médio no teste de levantar e sentar da cadeira foi de 21,7 segundos, a pontuação média da MIFm de 82,2 e da AIVDt de 21,2; 44,7% dos sujeitos apresentaram 1-2 critérios de fragilidade e 55,3% > 3 critérios. Houve associação significativa entre FM de MMII e independência funcional em função do número de critérios de fragilidade, sem homogeneidade em relação ao sexo e idade. A independência funcional sofreu influência significativa de sexo e FM de MMII.

**CONCLUSÕES:**

Os idosos com um ou dois critérios de fragilidade apresentaram melhor independência em todos os escores do teste de levantar e sentar da cadeira. Os sujeitos com maior FM de MMII apresentaram melhor independência funcional.

## INTRODUCTION

Sarcopenia, which comprises loss of muscle mass associated with aging, derives from a complex process.^1^ ^-^ ^3^ These changes to muscle composition result from muscle fiber atrophy, reduction in the production of certain hormones (testosterone, adrenal androgens and growth hormone) and inappropriate food intake, among other factors.^1^ ^-^ ^6^

The decrease in muscle strength resulting from sarcopenia causes significant functional loss with regard to performing activities of daily living (ADLs), and this is the main etiological factor in the development of functional dependence among elderly individuals.^1^ ^,^ ^3^ ^,^ ^6^ ^-^ ^12^ The association between muscle strength and functional independence has been pointed out in the literature, and the study by Janssen et al.^8^ deserves special attention. This assessed 504 elderly individuals and identified a significant correlation (P < 0.05) between sarcopenia and high risk of developing dependence for performing ADLs. 

Nevertheless, sarcopenia expressively contributes towards development of the frailty syndrome,^5^ ^,^ ^13^ ^-^ ^19^ which was defined by Fried and Walston^13^ as a state of physiological vulnerability, with diminishment of the capacity to maintain or recover homeostasis when faced with stress and/or environmental challenges. These authors proposed that frailty consisted of three elements: neuromuscular alterations, deregulation of the neuroendocrine system and dysfunction of the immunological system. Until the 1980s, frailty syndrome was understood as functional incapacity. However, studies have shown that these are distinct conditions, and that frailty can actually contribute towards development of functional dependence.^17^ ^-^ ^19^ In a study by Woods et al.,^19^ elderly women who were considered to be pre-frail and frail showed, respectively, 1.64 and 3.15 times greater chances of developing functional dependence, in comparison with non-frail individuals, after a three-year study. 

Most studies have shown that women are more likely to present functional incapacity and characteristics of the frailty phenotype, since they are more exposed and live longer than men.^14^ ^,^ ^20^ Studies on these differences are important for constructing strategies for elderly care. Considering that frailty and decreased muscle strength are associated with elderly people's functional abilities, the present study had the objectives of investigating the relationship between lower-limb muscle strength (LLMS) and functional independence, taking into consideration gender, age and a number of frailty criteria among elderly outpatients, and of investigating the influence of these variables on functional independence. 

## OBJECTIVE

The aims of this study were to compare lower-limb muscle strength (LLMS) with functional independence, taking into consideration gender, age and a number of frailty criteria, and to ascertain the influence of these variables on the independence of elderly outpatients.

## METHODS

This was a descriptive quantitative cross-sectional study, and data were gathered as part of a major project developed in the Geriatrics Outpatient Clinic of a university hospital in Campinas (Universidade Estadual de Campinas, Unicamp). It was approved by the institution's Research Ethics Committee, as decision no. 240/2003.

In this study, a non-probabilistic convenience sample of 150 elderly people of both sexes who were followed up as outpatients was assessed between October 2005 and October 2007. The subjects met the following inclusion criteria: agreeing to participate in the study, signing a free and informed consent statement and being in a cognitive condition for oral communication to be established, so that an interview could be conducted and the Mini-Mental State Examination could be applied, as prescribed by Bertolucci et al.^21^ The exclusion criteria were: refusal to participate, impaired oral communication, cognitive deficit that could harm comprehension and Mini Mental State Examination (MMSE) score lower than the cutoff score. 

As many older adults as possible per day were approached to inquire about their willingness to take part in the study, their availability for an interview and their compatibility with the inclusion criteria. We were able to interview two older adults a day.

The variables for this study were as follows: 

### Sociodemographic variables: gender and age; 

### Anthropometry: weight, height, body mass index (BMI) and handgrip strength; 

**Mobility and flexibility:** gait speed test and LLMS, which are part of the short physical performance battery (SPPB) that was proposed by Guralnik et al.^22^ and adapted to the Brazilian Portuguese language by Nakano;^23^

### Physical activity: practice and weekly frequency; 

**MMSE: **with cutoff score greater than or equal to 13 (for illiterate individuals), 18 (individuals with one to seven years of schooling) and 26 (individuals with eight years of schooling or more);^21^

**Depressive status: **two questions from the depression tracking scale (Center for Epidemiologic Studies Depression Scale, CES-D), which was developed by Radloff^24^ and validated in Brazil for the elderly population by Batistoni et al.^25^

To identify frailty, the criteria of Fried et al.^14^ were used with some adaptations, as follows:

- **Involuntary weight loss**: over the last year, over 4.5 kg or 10% of body weight;
- **Exhaustion:** self-reported fatigue assessed through two questions ("Did you feel that you had to make an effort to accomplish your habitual tasks?" and "Were you unable to do your things?") taken from the depression tracking scale CES-D. In the case of an affirmative answer for a period of three or more days of the previous week, the subject was graded as positive for exhaustion;
- **Decreased walking speed:** the time taken to walk a distance of 4.0 meters one way and 4.0 meters back again was measured using a chronometer, taking the best time for this course, adjusted according to sex and height. For men of heights < 1.73 and > 1.73 meters respectively, times of > 7 seconds and > 6 seconds were considered positive. For women of heights < 1.59 and > 1.59 meters respectively, times of > 7 seconds and > 6 seconds were considered positive;
- **Muscle weakness: **this was evaluated by means of a handgrip dynamometer. The individual under evaluation was positioned standing upright with the arms along the body, except for wheelchair users, who did the test in the seated position. The highest value was taken from three measurements of handgrip strength, with intervals of approximately five minutes between them, adjusted for sex and body mass index. Men were graded positive for muscle weakness when their handgrip strength was < 29.0 kgf for body mass index < 24.0 kg/m^2^; < 30.0 kgf for body mass index 24.1 to 26.0; and < 32.0 kgf for body mass index > 28.0. For women, the values were <17.0 kgf for body mass index < 23.0; < 17.3 kgf for body mass index 23.1 to 26.0; < 18.0 kgf for body mass index 26.1 to 29.0; and < 21.0 kgf for body mass index > 29.0.
- **Low level of physical activity:** this was graded positive for elderly individuals who were inactive or who performed physical activities less than twice a week.

After evaluating the frailty criteria, two groups were obtained: one group with one or two criteria (considered to be pre-frail) and the other with three or more criteria (considered to be frail), as suggested by Fried et al.^14^ All of the subjects in this study presented at least one of the criteria. 

To evaluate lower-limb muscle strength, the five-times sit-to-stand test (FTSST) was used. This forms part of the Short Physical Performance Battery instrument, which was proposed by Guralnik et al.^22^ and was adapted to the Brazilian Portuguese language by Nakano^23^ The test was undertaken using an armless chair that was 46 centimeters high from its seat to the floor. The individual under evaluation sat on the chair with arms folded across the chest. Points were awarded according to the time needed to complete the test. Zero was attributed when the elderly individual was unable to perform the test or required > 60 seconds to complete it; one point if the time needed was > 16.7 seconds; two points if the time was between 13.70 and 16.69 seconds; three points if the time was between 11.20 and 13.69 seconds; and 4 points if the time was < 11.19 seconds. Scores 3 and 4 for the lower-limb muscle strength test were grouped together because of the small number of subjects with each score (n = 8 and n = 6, respectively). 

Functional independence was assessed in relation to basic and instrumental activities of daily living activities (BADL and IADL), and was measured according to the motor score of the functional independence measurement (FIM)^26^ ^,^ ^27^ and the instrumental activities proposed by Lawton and Brody^28^ as adapted by Freitas and Miranda.^29^ FIM is an instrument containing 18 items, divided into two subscales: 1- motor FIM (mFIM), concerning self-care, sphincter control and mobility; 2- cognitive-social FIM, concerning communication and social cognition. Each item receives a score ranging from 1 (total dependence) to 7 (complete independence), with a total score ranging from 18 to 126. The motor component score ranges from 13 to 91 points. In assessing the instrumental activities, the following tasks were considered: food preparation; doing the household chores; washing and ironing the clothes; doing manual work; handling medication; using the telephone and handling money; doing the shopping and using means of transportation. If the task is performed "independently" three points are attributed; two points when there is "partial independence" and one point for "total dependence" and the total score ranges from 9 (maximum dependence) to 27 points (maximum independence). In both instruments, the higher the score is, the higher the functional independence is. 

The results were subjected to the following analyses. Descriptive analysis was performed to make measurements of position (mean, median, minimum and maximum) and dispersion (standard deviation). Cronbach's alpha coefficient was used to evaluate the reliability of the instruments, such that high internal consistency was indicated by values greater than or equal to 0.7. The Shapiro-Wilk test was used to point out situations of non-normal distribution of the sample, which would require subsequent use of nonparametric statistical tests. The Kruskal-Wallis test was used to compare the LLMS scores and FIM and IADL, taking into consideration sex, age and frailty criteria, followed by the post-hoc Dunn test. 

Univariate analysis of variance (ANOVA) was used to evaluate the impact of each variable of interest (sex, age, LLMS and frailty) on the scores of each functional independence evaluation tool (mFIM and IADL). Multivariate analysis of variance (MANOVA) was used to ascertain the combined influence of the variables of interest on the mFIM and IADL scores (dependent variables). Variables that were not normally distributed were transformed into ranks in these analyses. MANOVA was used to test the significance of the difference between measurements of two or more groups in relation to two or more dependent variables that were taken into account simultaneously. The significance level used was 5% (P < 0.05). 

## RESULTS

Table 1 shows that the mean age of the elderly individuals was 76.4 ± 7.8 years and that women predominated (64.2%).

**Table 1:** Sociodemographic, anthropometric and functional characterization of the individuals studied (n = 150)

Variable	Category	n (%)	Mean	SD	Median	Observed variation	Possible variation
Age (years)		150 (100.0)	76.4	7.8	76	60-93	
Sex	Female	96 (64.2)					
	Male	54 (35.8)					
Weight (kg)		150 (100.0)	63.2	14.4	60.2	34.6-106.5	
Height (cm)		149* (99.3)	154.6	8.9	154.0	135.0-181.5	
BMI (kg/m^2^)		149* (99.3)	26.4	5.5	25.8	15.4-41.6	
LLMS (seconds)		117^†^ (78.0)	21.7	7.9	20.3	8.1-60.3	
mFIM (*score*)		148* (98.7)	82.3	9.4	85.0	44-91	13-91
IADL (*score*)		149* (99.3)	21.2	4.9	22.0	9-27	9-27
Number of frailty criteria	1-2	67 (44.7)					
	3	83 (55.3)					

* one or two missing

† 26 subjects were unable to perform the tests and 77 were missing

BMI = body mass index

LLMS = lower limb muscle strength

FIM = functional independence measurement

IADL = instrumental activities of daily living

SD = standard deviation

The mean time taken to perform the LLMS test was 21.7 ± 7.9 seconds; 26 subjects were unable to perform the test; the mean mFIM score was 82.3 ± 9.4 and the mean IADL score was 21.2 ± 4.9. Regarding the frailty criteria, 67 individuals (44.7%) presented 1 to 2 criteria (and were considered to be pre-frail) and 83 (55.3%) presented 3 or more criteria (and were considered to be frail).

Reliability analysis on the tools was performed through calculating Cronbach's alpha coefficient, and this showed high internal consistency, with values of 0.92 for mFIM and 0.86 for IADL. 

Table 2 presents the results from comparisons between the median mFIM and IADL scores and the FTSST scores between the sexes. In the LLMS, men with scores of 3 and 4 presented significantly higher median mFIM (P < 0.001) and IADL (P = 0.001) than women with these scores in the LLMS. 

**Table 2:** Comparison between the lower limb muscle strength (LLMS) scores (five-times sit-to-stand test, FTSST), taking sex into consideration, and the median scores for the motor functional independence measurement (mFIM) and instrumental activities of daily living (IADL) among the elderly individuals studied (n = 150)

Variable	LLMS				P-value*
	0	1	2	3-4	
	Male/Female	Male/Female	Male/Female	Male/Female	
mFIM	82.5/75.0	88.5/84.0	88.0/88.0	90.0/83.0	< 0.001^(a)^
IADL	18.0/17.0	24.5/22.0	24.0/26.0	27.0/25.0	0.001^(b)^

* Kruskal-Wallis test for comparison of the variables according to muscle strength and sex, with Dunn post-hoc test

a 0/Fem ≠ 3-4/Male

b 0/Fem ≠ 3-4/Male

Fem = female sex

Male = male sex

Table 3 shows the comparison between FTSST and mFIM and IADL, taking age into consideration. There was a statistically significant difference between FTSST and the median mFIM (P = 0.016) among the elderly subjects aged 60 to 69 years and aged 80 years or over with LLMS score 0, in comparison with the subjects aged 80 years or over with LLMS scores of 3 and 4. Elderly individuals aged 80 years or over with FTSST scores of 3 and 4 showed higher median IADL (P < 0.001) in relation to the elderly individuals in the other age groups with FTSST score 0 and those aged 70 to 79 years with scores of 3 and 4. 

**Table 3:** Comparison between lower limb muscle strength (LLMS) scores (five-times sit-to-stand test, FTSST), taking age into consideration, and the median scores for the motor functional independence measurement (mFIM) and instrumental activities of daily living (IADL) among the elderly individuals studied (n = 150)

Variable	LLMS				P-value*
	0	1	2	3-4	
	60-69/70-79/ 80	60-69/70-79/ 80	60-69/70-79/ 80	60-69/70-79/ 80	
mFIM	68.0/81.0/77.5	88.0/87.0/85.0	85.5/88.0/84.0	83.5/83.0/89.5	0.016^(a)^
IADL	18.0/19.0/17.0	24.0/24.0/22.0	26.0/26.0/19.0	25.0/13.0/27.0	< 0.001^(b)^

* Kruskal-Wallis test for comparison of the variables according to muscle strength and age, with Dunn post-hoc test

a 0/60-69 ≠ 3-4/≥ 80 and 0/≥ 80 ≠ 3-4/≥ 80

b 0/≥ 80 ≠ 3-4/≥ 80, 3-4/70-79 ≠ 3-4/≥ 80, 0/70-79 ≠ 3-4/≥ 80, 2/≥ 80 ≠ 3-4/≥ 80 and 0/60-69 ≠ 3-4/≥ 80

Comparative analyses on median mFIM and IADL taking into consideration the number of frailty criteria according to the FTSST scores are presented in Table 4. Significantly higher median mFIM (P < 0.001) was observed for the elderly with LLMS score 1 and one or two frailty criteria, in comparison with the elderly individuals with LLMS scores of 0 and 3 and 4 with three or more frailty criteria. On the other hand, elderly individuals with LLMS score 0 and one or two frailty criteria presented significantly lower median mFIM (P < 0.001), in comparison with the elderly individuals with mFIM score 2 with three or more criteria. Elderly individuals with LLMS scores of 2, 3 and 4 and one or two criteria presented significantly higher median IADL (P < 0.001) than elderly individuals with LLMS scores of 0, 3 and 4 with three or more criteria. 

**Table 4:** Comparison between the lower limb muscle strength (LLMS) scores (five-times sit-to-stand test, FTSST), taking the number of frailty criteria into consideration, and the median scores for the motor functional independence measure (mFIM) and instrumental activities of daily living (IADL) among the elderly individuals studied (n = 150)

Variable	LLMS				P-value*
	0	1	2	3-4	
	1-2/ 3	1-2/ 3	1-2/ 3	1-2/ 3	
mFIM	76.0/80.0	89.0/85.0	88.0/89.0	87.0/80.0	< 0.001^(a)^
IADL	20.0/17.0	24.0/23.0	25.0/24.0	25.0/13.0	< 0.001^(b)^

* Kruskal-Wallis test for comparison of the variables according to muscle strength and frailty criteria, with Dunn post-hoc test

a 0/≥ 3 ≠ 1/1-2, 0/≥ 3 ≠ 2/≥ 3, 0/1-2 ≠ 1/1-2, 0/1-2 ≠ 2/≥ 3, 3-4/≥ 3 ≠ 1/1-2 and 3-4/≥ 3 ≠ 2/≥ 3

b 3-4/≥ 3 ≠ 2/1-2, 3-4/≥ 3 ≠ 3-4/1-2, 0/≥ 3 ≠ 2/1-2 and 0/≥ 3 ≠ 3-4/1-2.

Considering that LLMS is a variable that presents a statistically significant relationship with mFIM and IADL scores, taking into consideration age, sex and frailty criteria, a variance analysis model was created with the aim of identifying the variables that influenced functional independence the most. Thus, by analyzing the impact of each variable on functional independence (Table 5), it was observed that mFIM was significantly influenced by sex (P = 0.004) and LLMS (P = 0.010). IADL was influenced by sex (P = 0.044), age (P = 0.023) and LLMS (P = 0.019). Hence, men and subjects with higher LLMS presented better mFIM and IADL scores; the younger elderly individuals presented better mFIM and IADL scores and the younger elderly individuals also presented better IADL scores. The frailty criteria did not have any influence on functional independence. In the multivariate analysis (MANOVA) presented in Table 5, independence assessed by means of mFIM and IADL scores continued to be significantly influenced by the variables of sex (P = 0.015) and LLMS (P = 0.026).

**Table 5:** Results from multivariate analysis of variance (MANOVA) and univariate analysis of variance (ANOVA)

Variables	MANOVA	mFIM	ANOVA
	P-value		IADL
Sex	0.015	0.004*	0.044*
Age	0.063	0.270*	0.023*
LLMS	0.026	0.010*	0.019*
Frailty	0.196	0.219*	0.071*

* P-value

mFIM = functional independence measurement

IADL = instrumental activities of daily living

LLMS = lower-limb muscle strength

## DISCUSSION

The main results showed that functional independence was positively related to lower-limb muscle strength at different frailty levels. The sociodemographic characteristics of the elderly individuals attended at the outpatient clinic studied corresponded to the average profile for Brazilian elderly people, i.e. the group was formed mostly by women and was close to the mean age of the elderly Brazilian population (73.1 years).30 A study using the same sample found that 71.2% of the volunteers had five or more diseases and that 67.1% had five or more comorbidities.^31^ Regarding frailty characteristics, a community-based study in the same city showed that females, higher age groups and lower income groups correlated with greater frailty, with more fatigue, less muscle strength and slower gait.^32^

Previous studies^33^ ^-^ ^40^ that used the FTSST to measure LLMS among elderly individuals showed higher performance than was observed in the present study, in which the mean time taken in the FTSST was 21.7 seconds. It might be possible to explain this discrepancy in terms of the profile of elderly individuals attended at the outpatient clinic studied, which followed certain inclusion criteria, such as advanced age and functional deficit. A study by Aslan et al.^39^ on 115 elderly outpatients who were able to walk without help and did not present any neurological disease or visual and/or auditory problems showed that the mean time taken to perform the FTSST was 14.4 ± 6.88 seconds. In a study on 44 elderly individuals of mean age 83.13 ± 3.3 years who were able to walk with or without a device to help them and who presented clinical stability, Ferreira et al.^38^ found that the mean time taken to perform the FTSST was 12.72 ± 6.94 seconds. Although these elderly individuals presented advanced age, one of the inclusion criteria of their study was that the subjects needed to be able to walk. On the other hand, this criterion was not taken into consideration in the methodology of the present study. 

In comparing LLMS and functional independence in relation to sex, age and number of frailty criteria, it was found that the FTSST distinguished the degree of independence between men and women only at extreme scores, with greater independence among the men. Previous studies^35^ ^,^ ^41^ ^-^ ^43^ on elderly outpatients living in the community did not show any difference in performance in the FTSST between the sexes, except for the study by Barbosa et al.^20^ in which the influence of higher numbers of diseases and greater obesity presented by women would explain their worse performance in the test, in comparison with the men. In the present study, the elderly individuals of both sexes probably presented similar performance in the FTSST, thus corroborating the findings in literature, which can explain why there was a difference in independence between the sexes only at the minimum and maximum scores of the test. 

Comparing LLMS and independence among the age groups, there was only a significant difference at the minimum and maximum scores, with greater independence among the subjects aged 80 years or over and with higher LLMS. In the literature,^35^ ^,^ ^37^ ^,^ ^38^ significant associations between FTSST and age have been reported, with lower performance in the test among older elderly individuals. It is likely that because of the profile of the elderly individuals attended at the Geriatric Outpatient Clinic studied here, where the outpatients are aged between 60 and 79 years, some degree of functional impairment was presented. All the subjects studied may have presented similar performance in the test, independently of age, and this possibly explains the finding that there was only a difference between the age groups at the extremities of the test scores, and might also explain the better performance of the elderly individuals aged 80 years or over. It was also found that the FTSST significantly distinguished the functional independence of the elderly individuals with one or two frailty criteria from those with three or more criteria, except for the individuals with LLMS score 0 and one or two criteria in relation to those with LLMS score 2 and three or more criteria, in which the latter presented significantly higher mFIM scores. It is possible that the individuals with one or two frailty criteria presented criteria relating to muscle strength (grip strength, gait speed and physical activity) that might have interfered with their BADL performance.

Concerning functional independence, it was found that sex and LLMS influenced BADL and IADL performance: these results are consistent with reports in the literature, except for the absence of the influence of age.^43^ ^-^ ^56^ Guralnik et al.^51^ studied 1,122 elderly individuals and also found that the subjects with higher LLMS evaluated through FTSST presented greater functional independence, a conclusion that was obtained after four years of study. The study by Rosa et al.^47^ which investigated the determining factors of functional independence among elderly individuals, identified that females and individuals with advanced age presented greater probability of developing functional dependence (P < 0.001). Again, it is likely that the absence of influence of age on independence in the present study was due to the profile of the elderly individuals attended at the Geriatric Outpatient Clinic. 

There are some limitations to the present study. Because of the absence of a group of elderly people without frailty criteria, other comparisons were not possible with the non-frail individuals. This is once again possibly due to the profile of the elderly individuals followed up at the Geriatrics Outpatient Clinic. In general, these elderly people seek this health service because they already have some health care needs. In addition, the elderly individuals of this sample present peculiar characteristics; therefore, the results cannot be generalized for the population in general. 

Nonetheless, it was possible to identify significant differences in lower-limb muscle strength between the groups with one or two frailty criteria and those with three or more criteria, thus showing that these aspects should be taken into consideration in developing actions aimed towards improving elderly people's quality of life. 

The findings from our study draw attention to the role of decreased muscle strength among elderly people in relation to development of functional dependence, thus suggesting that recovery and rehabilitation programs conducted by qualified professionals need to be created. Further research should evaluate the relationship between frailty and functional performance in settings other than outpatient clinics, especially among women.

## CONCLUSION

It was observed that male elderly individuals with advanced age (> 80 years), with one or two frailty criteria and higher LLMS presented better functional independence than younger female elderly individuals with lower LLMS and three or more frailty criteria. Separately, men and elderly individuals with higher LLMS presented better functional independence.
